# Sensitivity Optimization of Surface Acoustic Wave Yarn Tension Sensor Based on Elastic Beam Theory

**DOI:** 10.3390/s22239368

**Published:** 2022-12-01

**Authors:** Yong Ding, Lili Gao, Wenke Lu

**Affiliations:** 1 School of Information Science and Technology, Donghua University, Shanghai 201620, China; 2 School of Network and Communication Engineering, Jinling Institute of Technology, Nanjing 211169, China

**Keywords:** yarn tension sensor, SAW, simply supported beam, elastic beam theory, sensitivity

## Abstract

The measurement of yarn tension has a direct impact on the product quality and production efficiency in the textile manufacturing process, and the surface acoustic wave (SAW) yarn tension sensor is a good option for detecting the yarn tension. For SAW yarn tension sensors, sensitivity is an important indicator to assess their performance. In this paper, a new type of SAW yarn tension sensor based on a simply supported beam structure is studied to improve the sensitivity of the fixed beam SAW yarn tension sensor. The sensitivity analysis method based on elastic beam theory is proposed to illustrate the sensitivity optimization. According to the analysis results, the sensitivity of the SAW yarn tension sensor can be greatly improved by using a simply supported beam structure compared to the s fixed beam structure. Moreover, from the calibration experiment, the sensitivity of the simply supported beam SAW yarn tension sensor is 2.5 times higher than that of the fixed beam sensor.

## 1. Introduction

The measurement of yarn tension is very important in the textile manufacturing process, which directly affects the product quality, the production efficiency, and the subsequent processing [1,2,3,4]. Significantly, with the rapid development of SAW [5,6,7,8,9,10] devices, SAW sensors [11,12,13] are being used in a wide range of industries. Furthermore, the SAW yarn tension sensor studied in [14,15,16,17,18,19] is a good selection to adapt to today’s textile manufacturing; it has excellent characteristics, such as high stability, high sensitivity, fast response speed, strong anti-interference ability, small size, low cost, and so on [20].

A cantilever beam SAW yarn tension sensor is introduced in [21], and the reliability of the sensor is poor because of the cantilever beam, which is easy to break during the measuring process [22], and this is disadvantageous to the universality of the sensor in practical applications. A fixed beam SAW yarn tension sensor is discussed in [22], and it was concluded that the sensitivity of the fixed beam SAW yarn tension sensor in [22] is 12.996 kHz/N, which is much lower than that of the cantilever beam sensor. It is well known that the lower the sensitivity of the yarn tension sensor, the poorer the quality of the yarn and fabric product, and the lower the yield of the yarn and fabric product [15]. There is no doubt that the fixed beam sensor studied in [20,22] holds important value for the promotion of the SAW yarn tension sensor in practical applications. However, the improvement of the sensitivity of the fixed beam sensor is a key problem that needs to be solved urgently.

From [15,22], the size of the piezoelectric substrate and the position of the interdigital transducer (IDT) are key factors affecting the sensitivity of the SAW yarn tension sensor, which are determined based on the simulation results of the finite element method (FEM) [23,24]. It is obvious that the sensitivity analysis of the SAW yarn tension sensor is overly dependent on the complex simulation methods. There still lacks a simple and efficient analysis method for sensitivity optimization. For the fixed beam SAW yarn tension sensor, the sensitivity of the sensor can be improved by increasing the length of the piezoelectric substrate. However, in practical applications, the SAW yarn tension sensor generally needs to be installed in textile machinery with limited space, which makes it difficult to increase the size of the sensor to improve the sensitivity.

In this paper, the bending moment distribution analysis of a beam based on elastic theory is proposed as a theoretical analysis method for the sensitivity analysis of the SAW yarn tension sensor, which is simple and efficient compared with the simulation methods. Targeting the sensitivity enhancement of the fixed beam sensor, a new type of SAW yarn tension sensor based on the simply supported beam [25,26,27,28] structure is adopted in this paper. According to the analysis results, it can be concluded that the sensitivity of the SAW yarn tension sensor will be significantly improved when the structural form of the beam is changed from a fixed beam to a simply supported beam, without increasing the size of the SAW sensor. Moreover, the experimental verification based on test data is elaborated in this paper. By comparison, the sensitivity of the simply supported beam SAW yarn tension sensor studied in this paper is about 2.5 times that of the fixed beam sensor.

## 2. Principle Analysis

### 2.1. The Working Principle of SAW Yarn Tension Sensor

**Figure 1:**
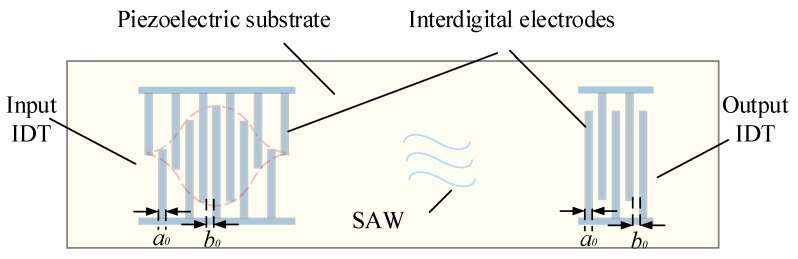
Structure diagram of the SAW sensor.

**Figure 2:**
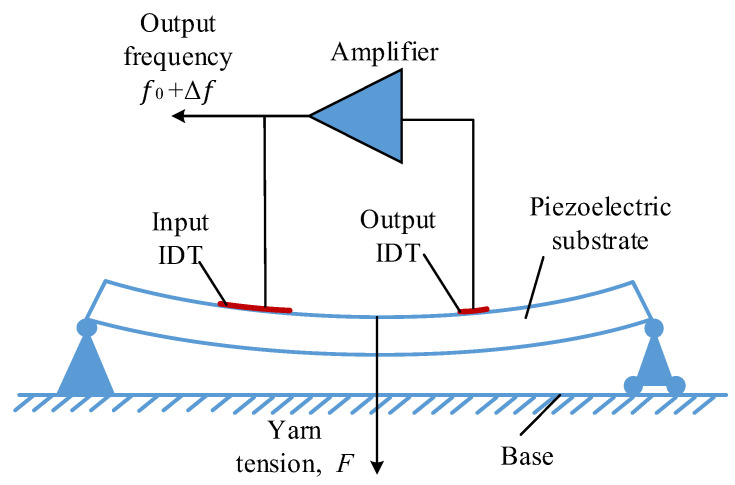
The working principle diagram of the SAW yarn tension sensor.

The structure diagram of the SAW sensor is shown in Figure 1, and Figure 2 shows the working principle of the SAW yarn tension sensor. From Figure 1, the IDTs are positioned on the upper surface of the piezoelectric substrate, *a*_0_ represents the width of the interdigital electrodes, when the yarn tension, *F* = 0, *b*_0_ represents the corresponding interdigital electrodes spacing, and *a*_0_ = *b*_0_. The relationship between the output frequency, *f*_0_ of the SAW oscillation (when the yarn tension, *F* = 0) and the SAW wavelength, *λ*_0_ is as follows:

(1) $$f_{0}=\frac{v_{0}}{\lambda_{0}}=\frac{v_{0}}{2\left(a_{0}{+b}_{0}\right)}=\frac{v_{0}}{{4b}_{0}}$$

where *λ*_0_ = 2 (*a*_0_ + *b*_0_) = 4*b*_0_; and *v*_0_ represents the SAW propagation speed when the yarn tension *F* = 0.

As can be seen from Figure 2, the yarn tension *F* loaded on the piezoelectric substrate causes bending deformation of the piezoelectric substrate. The change in the yarn tension *F* causes a change in the interdigital electrodes spacing, as well as in the propagation speed of the SAW [16]. From Equation (1), the output frequency of the SAW oscillation varies with the yarn tension, *F*. So, the measurement of yarn tension, *F* can be realized by detecting the frequency variation, Δ*f*.

### 2.2. Sensitivity Analysis of SAW Yarn Tension Sensor Based on Elastic Beam Theory

**Figure 3:**
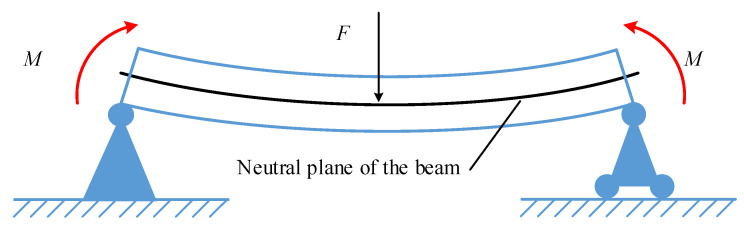
Schematic diagram of elastic deformation of the beam.

For the SAW yarn tension sensor, the piezoelectric substrate is equivalent to a slender beam, and the yarn tension to be measured corresponds to a concentrated load, *F*, acting on the beam. From elastic beam theory, when *F* is loaded within the elastic range, the beam undergoes elastic deformation [29] under the action of bending moment *M*, as shown in Figure 3.

**Figure 4:**
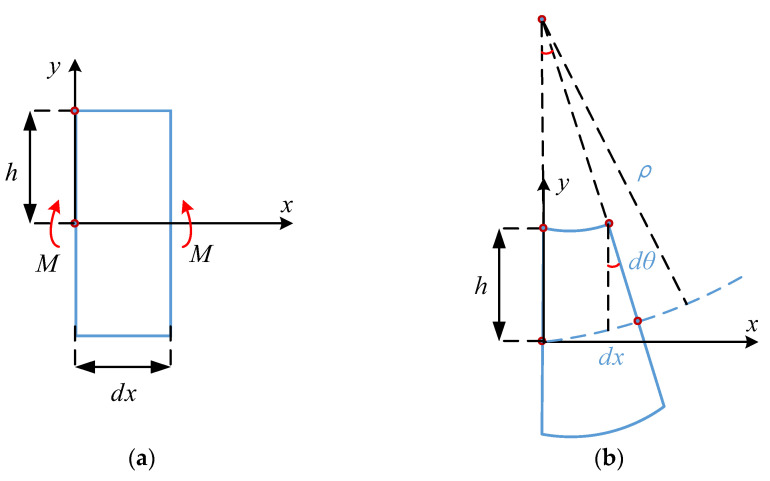
Deformation of micro-segment beam: (**a**) micro-segment of a beam; and (**b**) deformation diagram.

From Figure 4a, a micro-segment of length *dx* is cut from the beam, shown in Figure 3, using two adjacent cross-sections. The deformation diagram of the micro-segment beam is shown in Figure 4b. Suppose that after the beam is bent (under the action of bending moment *M*), the two cross-sections remain in plane but are turned around their respective neutral planes by an angle *dθ*. In the coordinate system of Figure 4, the *x*-axis is along the axis of the beam and the *y*-axis is along the height of the cross-section. In Figure 4b, the length variation, Δ*dx*, of the upper surface (height from the neutral plane is equal to *h*) of the micro-segment is as follows:

(2)  Δdx=-hdθ

where *h* is equal to half the height of the beam, and the negative sign indicates the occurrence of the compression deformation.

The expression for the strain *ε* in the upper surface of the micro-segment is as follows:

(3) $$\varepsilon =\frac{\Delta \mathrm{dx}}{\mathrm{dx}}$$

From Equations (2) and (3):

(4) $$\varepsilon =\frac{\Delta \mathrm{dx}}{\mathrm{dx}}=-h\frac{d\theta }{\mathrm{dx}}=-\frac{h}{\rho }$$

From elastic beam theory, there is an expression [29]:

(5) $$\frac{1}{\rho }=\frac{M}{\mathrm{EI}}\;$$

in Equations (4) and (5), *ρ* is the radius of curvature of the axis after bending; *M* is the bending moment and is a function of cross-section position *x*; and *EI* is the bending stiffness constant.

In this paper, the key problem is to improve the sensitivity of the fixed beam SAW yarn tension sensor. The sensitivity of the SAW yarn tension sensor reflects the change of sensor output frequency under unit tension [20]. According to the working principle of the SAW yarn tension sensor, the yarn tension causes elastic deformation of the piezoelectric substrate, which changes the center frequency of the SAW sensor; the expression is as follows [22]:

(6) $${\Delta f=f}_{0}\frac{(k^{\prime }-1)\varepsilon }{1+\varepsilon }$$

where *f*_0_ represents the output frequency of the SAW sensor (when the yarn tension, *F* = 0); *k′* is the elastic stiffness constant of the piezoelectric substrate [22]; *ε* represents the strain of the piezoelectric substrate when the yarn tension *F* ≠ 0; and Δ*f* is the corresponding frequency variation.

From Equation (6), under the action of tiny yarn tension, the strain, ε ≪ 1, which gives [16]:

(7) $${\Delta f=f}_{0}\;(k^{\prime }-1)\varepsilon$$

As for the SAW yarn tension sensor, the position of the IDTs on the upper surface of the piezoelectric substrate directly affects the sensitivity of the sensor [16,22]. From Equation (6), it is obvious that the sensitivity of the SAW yarn tension sensor has a linear relationship with the strain of the piezoelectric substrate (IDTs placed part), under the same yarn tension.

Combining Equations (4), (5) and (7) yields:

(8) $${|\Delta f|=|\mathrm{hf}}_{0}\left(K-1)\right|\frac{\left|M\right|}{\mathrm{EI}}$$

As can be seen from Equation (8), the sensitivity of the SAW yarn tension sensor is linearly related to the absolute value of the bending moment of the beam (IDTs placed part), under the same yarn tension.

Therefore, this paper proposes a new type of SAW yarn tension sensor based on a simply supported beam structure that has a larger bending moment maximum compared with the fixed beam structure in [22] under the same yarn tension, and thus improves the sensitivity of the SAW yarn tension sensor. The comparative analysis of the bending moment distribution of these two types of beam structures, based on elastic beam theory, is shown in the next section.

## 3. Sensitivity Optimization Based on Elastic Beam Theory

**Figure 5:**
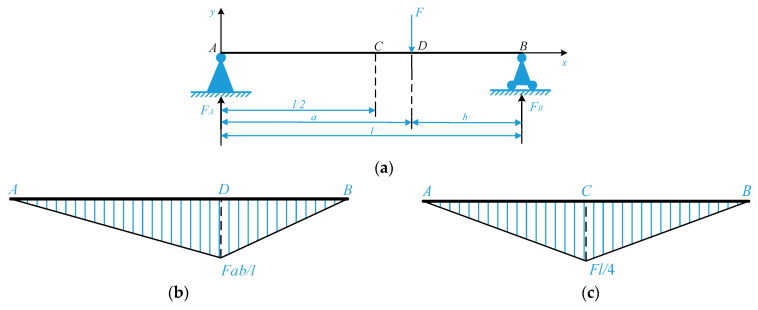
Schematic diagram of simply supported beam and bending moment diagram: (**a**) the simply supported beam; (**b**) the bending moment diagram; and (**c**) the bending moment diagram (when *F* is loaded at the midpoint of the beam).

The simply supported beam *AB* (*a ≥ b*) is shown in Figure 5a, and the solution process of its bending-moment equation is as follows:

According to the static balance method, *F_A_* and *F_B_* are the binding forces of the beam at supports *A* and *B*, as follows:

(9) $$F_{A}=\frac{\mathrm{Fb}}{l}{\;,\;F}_{B\;}=\frac{\mathrm{Fa}}{l}$$

Since there is a concentrated force, *F* at point *D* of the beam, it is necessary to divide it into part *AD* and part *DB* to establish the bending moment equation, respectively, i.e.:

$$M_{1}\left(x\right)=\frac{\mathrm{Fb}}{l}x\;,\;0\leq x\leq a\;;$$

(10) $$M_{2}\left(x\right)=\frac{\mathrm{Fb}}{l}x\;-\;F\left(x-a\right)\;,\;a\;\leq \;x\;\leq \;l$$

where *M*_1_(*x*) and *M*_2_(*x*) represent the bending moment equations of part *AD* and part *DB*, respectively.

The bending moment diagram of the simply supported beam *AB* is shown in Figure 5b. According to Equation (10) or the bending moment diagram of a simply supported beam, it is obvious that the maximum, *M*_1max_, is as follows:

(11) $$M_{1\max\;}{=M}_{1}\left(x\right){|}_{x=a}=\frac{\mathrm{Fab}}{l}$$

From Equation (11), when *a = b = l/2*, i.e., the concentrated load *F* acts on the midpoint of the beam, the bending moment of the simply supported beam *AB* reaches the maximum value, |*M_AB_*|*_max_*, as follows, and the maximum bending moment occurs at the midpoint of the beam; the bending moment diagram is shown in Figure 5c:

(12) $${|M}_{\mathrm{AB}}{|}_{\max}=\frac{\mathrm{Fl}}{4}$$

Therefore, for the simply supported beam SAW yarn tension sensor, the maximum sensitivity can be obtained when the IDTs are placed at the midpoint of the upper surface of the piezoelectric substrate and the yarn tension is loaded at the midpoint of the piezoelectric substrate.

**Figure 6:**
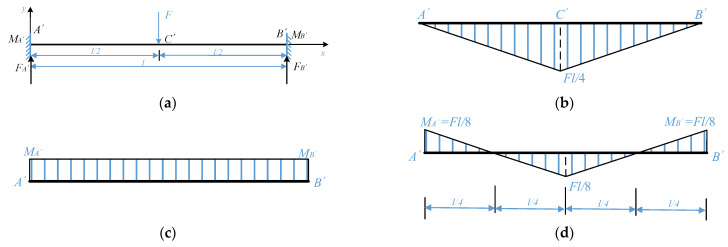
Schematic diagram of fixed beam and bending moment diagrams: (**a**) the fixed beam; (**b**) the free bending moment diagram; (**c**) the fixed-end moment diagram; and (**d**) the resultant bending moment diagram.

As for the fixed beam SAW yarn tension sensor studied in [22], the concentrated load (yarn tension) is loaded at the midpoint of the piezoelectric substrate. For comparison, this paper only discusses the special case that the concentrated load, *F* acts on the midpoint of the fixed beam, as shown in Figure 6a.

In Figure 6a, *M_A′_* and *M_B′_* represent the fixed-end moments at the ends *A′* and *B′* of the fixed beam *A′B′*, respectively, and *F_A′_* and *F_B′_* are the binding forces of the beam at supports *A′* and *B′*. From symmetry and the static balance method:

(13) $$M_{A^{\prime }}{=M}_{B^{\prime }}{\;,\;F}_{A^{\prime }}{=F}_{B^{\prime }}=\frac{F}{2}$$

According to the elastic beam theory, the fixed beam *A′B′* may be regarded as a simply supported beam carrying a concentrated load, *F*, at the midpoint of the beam and with the moments *M_A′_* and *M_B′_* applied at the supports *A′* and *B′*. The bending moment diagrams corresponding to these two loading cases are shown in Figure 6b,c and are termed the free bending moment diagram and the fixed-end moment diagram [29], respectively.

According to the moment-area method [29], the area of the free bending moment diagram is numerically equal to the area of the fixed-end moment diagram. From Figure 6b,c:

(14) $$M_{A^{\prime }}l=\frac{1}{2}\frac{\mathrm{Fl}}{4}l\;$$

which gives:

(15) $$M_{A^{\prime }}{=M}_{B^{\prime }}=\frac{\mathrm{Fl}}{8}$$

According to the superposition principle [29], the resultant bending moment diagram of the fixed beam *A’B’* can be obtained, as shown in Figure 6d.

From the resultant bending moment diagram, it is obvious that there is a maximum bending moment, |*M_A’B’_*|*_max_*, of the fixed beam *A’B’* at the midpoint of the beam, as follows:

(16) $${|M}_{A^{\prime }B^{\prime }}{|}_{max}=\frac{\mathrm{Fl}}{8}$$

Therefore, for the fixed beam SAW yarn tension sensor, in the case that the yarn tension is loaded at the midpoint of the piezoelectric substrate, the maximum sensitivity can be obtained when the IDTs are placed at the midpoint of the piezoelectric substrate.

**Figure 7:**
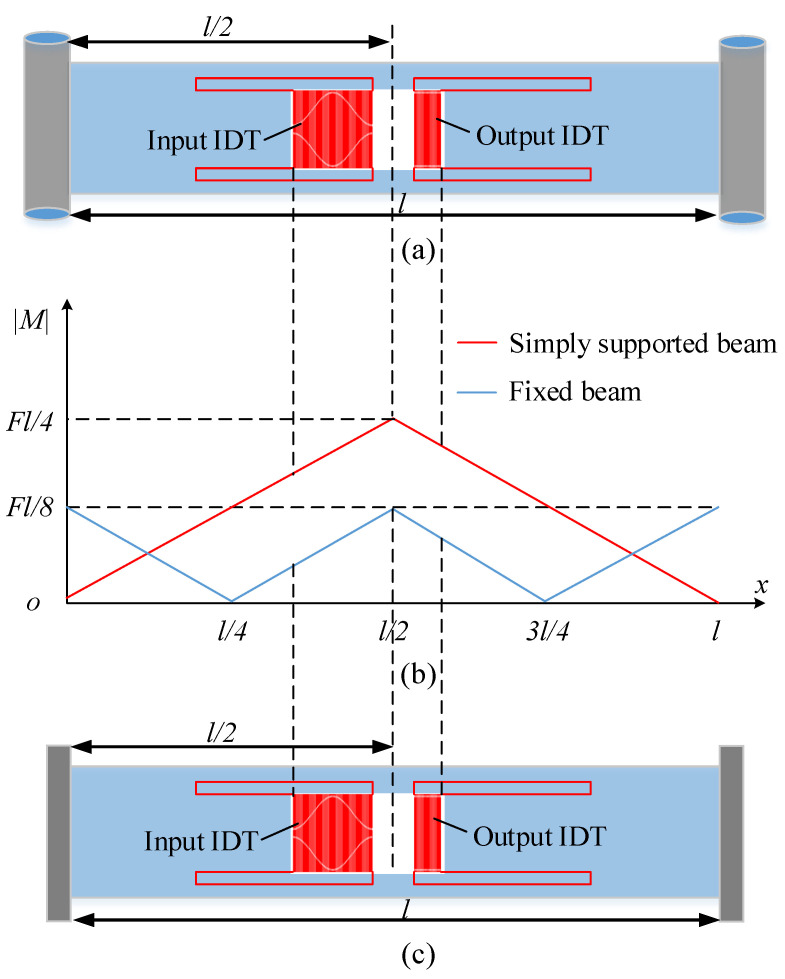
The analysis results of sensitivity optimization based on the elastic beam theory: (**a**) structure diagram of the piezoelectric substrate of the simply supported beam SAW yarn tension sensor; (**b**) distribution diagram of the absolute value of bending moment; and (**c**) structure diagram of the piezoelectric substrate of the fixed beam SAW yarn tension sensor.

The analysis results of the sensitivity optimization based on the elastic beam theory is shown in Figure 7. Figure 7a shows the structure diagram of the piezoelectric substrate of the simply supported beam SAW yarn tension sensor designed in this paper. The structure diagram of the piezoelectric substrate of the fixed beam SAW sensor, introduced in [22], is shown in Figure 7c. Moreover, Figure 7b shows the distribution diagram of the absolute value of the bending moments of the beams. As can be seen from Figure 7b, under the same yarn tension, the absolute value of the bending moment of the piezoelectric substrate (IDTs placed part) of the simply supported beam SAW yarn tension sensor studied in this paper is higher than that of the fixed beam sensor across the board, which means that the sensitivity of the simply supported beam SAW sensor will be significantly higher than that of the fixed beam sensor.

Through the above description, the sensitivity optimization method based on the elastic beam theory adopted in this paper can transform the sensitivity analysis of the sensor into the solution of the bending moment distribution of the beam structure, which is simple and efficient.

## 4. The Experimental Verification

### 4.1. Design of the Simply Supported Beam SAW Sensor

**Figure 8:**
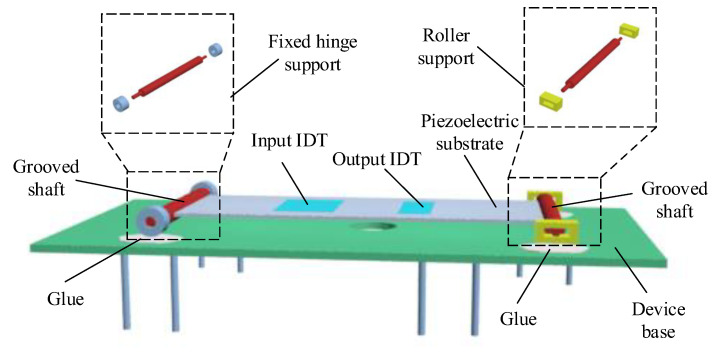
Model diagram of simply supported beam SAW sensor.

Figure 8 shows the model diagram of the simply supported beam SAW yarn tension sensor; it can be seen that the simply supported beam structure is composed of a fixed hinge support, a rolling support, and a piezoelectric substrate.

For comparison purposes, the main design parameters of IDTs and piezoelectric substrate of the simply supported beam SAW sensor designed in this paper are the same as those of the fixed beam sensor. The IDTs with an unbalanced split electrode geometry were used in order to suppress the finger reflection effects, and the electrode-overlap envelope of the input IDT was weighted according to the Hamming function [22]. The main design parameters are shown in Table 1.

### 4.2. Experiment and Result Analysis

**Figure 9:**
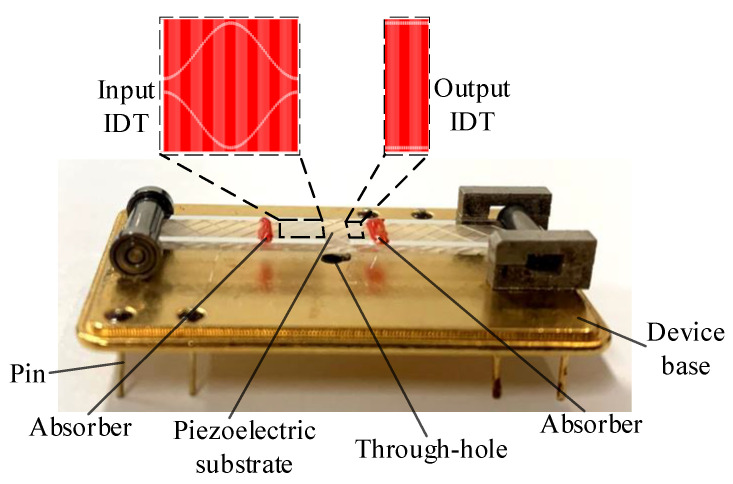
The simply supported beam SAW yarn tension sensor.

**Figure 10:**
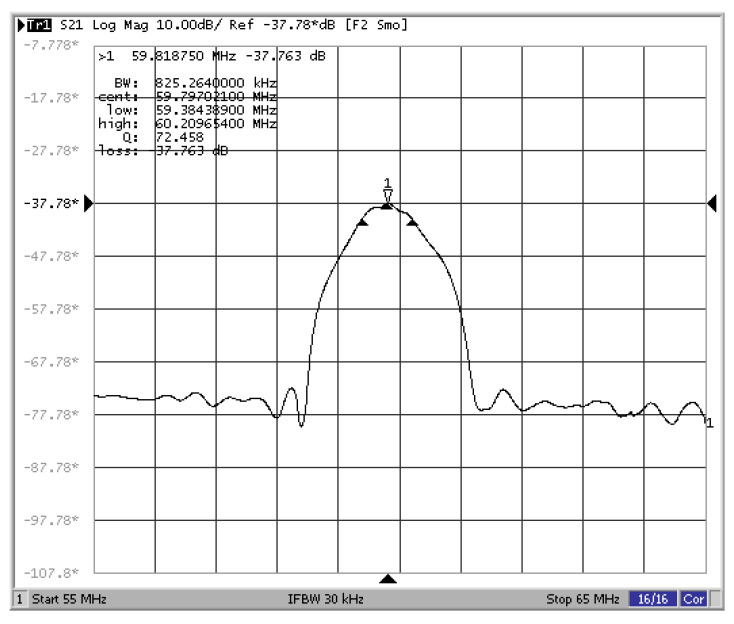
Frequency characteristics of the simply supported beam SAW sensor (no force on the piezoelectric substrate, 25 °C).

The picture of the simply supported beam SAW yarn tension sensor is shown in Figure 9. The frequency characteristics of the simply supported beam SAW sensor are shown in Figure 10. The center frequency of the SAW sensor at a temperature of 25 °C is about 59.797 MHz when the tension is equal to 0 N.

**Figure 11:**
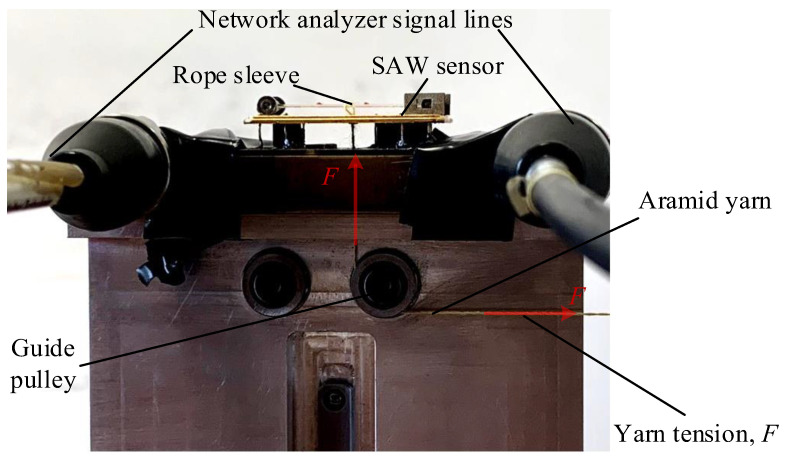
The measurement system of simply supported beam SAW yarn tension sensor.

In this paper, the calibration experiment on the relationship between yarn tension *F* and the frequency variation Δ*f* is performed. Figure 11 shows the measurement system of simply supported beam SAW yarn tension sensor. As can be seen from Figure 11, the yarn tension to be measured is loaded at the midpoint of the piezoelectric substrate by a rope sleeve and a guide pulley, and the output frequency of the sensor is detected by a network analyzer. In [22], the yarn tension is loaded on the piezoelectric substrate by a connecting rod. In this paper, the Aramid yarn with high modulus, stable size, and low shrinkage is used to replace the connecting rod, which reduces measurement errors and is more reliable.

The yarn tension was tested in the range of 0–1 N at an ambient temperature of 25 °C. The experimental data of the calibration experiment are shown in Table 2. According to the least square fitting technique [30], the fitting linear equation between yarn tension *F* and the frequency variation Δ*f* is as follows:

(17) Δf=32.822 F - 0.0959

**Figure 12:**
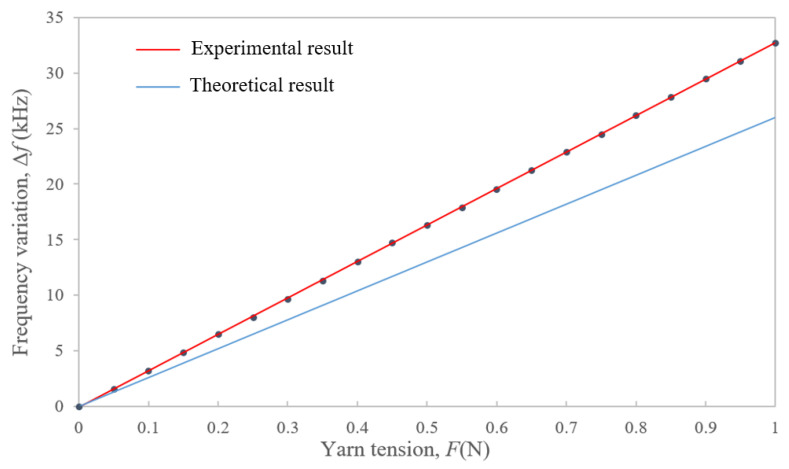
Sensitivity analysis results of the simply supported beam SAW yarn tension sensor.

From Equation (17), the experimental result of the sensitivity of the simply supported beam SAW yarn tension sensor is 32.822 kHz/N, which is 2.5 times higher than that of the fixed beam sensor (in [22], the sensitivity of the fixed beam SAW yarn tension sensor is 12.996 kHz/N). According to the theoretical analysis result, the sensitivity of the simple supported beam SAW yarn tension sensor is theoretically twice that of the fixed beam sensor (i.e., the theoretical result of the sensitivity of the simple supported beam SAW yarn tension sensor is 25.992 kHz/N). The results are shown in Figure 12. Because of the optimization of the measurement system, the experimental result of the sensitivity of the simply supported beam SAW yarn tension sensor is better than the theoretical result.

## 5. Conclusions

For the sensitivity enhancement of the fixed beam SAW yarn tension sensor, a new type of SAW yarn tension sensor based on simply supported beam is studied in this paper. The sensitivity analysis method based on elastic beam theory is proposed in this paper to illustrate the sensitivity difference between SAW yarn tension sensors with different structural forms, which is simple and efficient and can be used as an analysis tool for the sensitivity optimization of the SAW yarn tension sensors. According to the analysis results, the sensitivity of the SAW yarn tension sensor can be greatly improved by using a simply supported beam structure compared to the s fixed beam structure, without changing the size of the SAW sensor.

From the calibration experiment, it can be summarized that the sensitivity of simply supported beam SAW yarn tension sensor is 2.5 times higher than that of the fixed beam SAW yarn tension sensor. The research content of this paper illustrates the feasibility of the simply supported beam SAW yarn tension sensor in terms of sensitivity enhancement, which also contributes to the optimization and promotion of the SAW yarn tension sensor.

In practical applications, the stability of the simply supported beam SAW yarn tension sensor relies heavily on a rational structural design (the implementation of the fixed hinge support and the rolling support) and a sophisticated manufacturing process. In addition, the application to some textile machinery with limited space requires more efforts in the further integration and miniaturization of the sensor.

## Figures and Tables

**Table 1:** The main design parameters of the simply supported beam SAW sensor.

Piezoelectric Substrate	Material	Quartz
	Size	Length = 30 mm, Width = 6 mm, Height = 0.5 mm
IDTs	Electrode material	Aluminum
	Central frequency of the design	60.0 MHz
	Pairs number of interdigital bars in the input IDT	72
	pairs number of interdigital bars in the output IDT	24
	Distance between input IDT and output IDT	2 mm
	Width of interdigital bar	3.289583 μm, 9.868750 μm
	Spacing of interdigital bars	6.579167 μm
	Wavelength	52.633333 μm

**Table 2:** The data of the calibration experimental.

*F* (N)	0	0.05	0.10	0.15	0.20	0.25	0.30
Δ*f* (kHz)	0	1.568	3.233	4.867	6.464	8.018	9.626
*F* (N)	0.35	0.40	0.45	0.50	0.55	0.60	0.65
Δ*f* (kHz)	11.327	13.015	14.725	16.323	17.91	19.573	21.226
*F* (N)	0.70	0.75	0.80	0.85	0.90	0.95	1.00
Δ*f* (kHz)	22.912	24.491	26.191	27.879	29.478	31.08	32.714

## Data Availability

The data reported in this manuscript is accessible based on reasonable requests to the corresponding author.
